# Editorial: Role of induced pluripotent stem cells (iPSCs) in regenerative medicine, disease modeling and drug discovery

**DOI:** 10.3389/fbioe.2025.1734717

**Published:** 2025-12-04

**Authors:** Kim C. O’Connor, Hayden J. Stanley, Bruce A. Bunnell, Raffaele De Caro

**Affiliations:** 1 Department of Chemical and Biomolecular Engineering, Tulane University, New Orleans, LA, United States; 2 Department of Microbiology, Immunology, and Genetics, University of North Texas Health Science Center, Fort Worth, TX, United States; 3 Section of Human Anatomy, Department of Neuroscience, University of Padova, Padua, Italy

**Keywords:** IPSC (induced pluripotent stem cells), personalize cell therapy, disease modeling, drug discovery, regenerative medicine

Yamanaka and Takahashi’s groundbreaking research on induced pluripotent stem cells (iPSCs) fundamentally transformed the field of regenerative medicine (Takahashi and Yamanaka, 2006). Genetically reprogramming somatic cells to a pluripotent state provides a virtually unlimited supply of any cell type in the body from a patient. Since their discovery in 2006, iPSCs have enabled remarkable advances in personalized cell therapies, disease models, and drug development (Nicholson et al., 2022). Yet there are still significant challenges to realizing the full potential of iPSC technology. This Research Topic highlights recent efforts to overcome some of these barriers to achieve more viable, functionally mature and effective iPSCs.

Cryostorage is essential for the transportation and long-term preservation of iPSCs in research and clinical applications. The viability and function of stem cells are threatened by transient warming events at any step along the cold chain from source to destination (Pogozhykh et al., 2017). Temperature fluctuations commonly occur when cells are moved from long-term storage in the vapor phase of liquid nitrogen at −150 °C to dry ice at −80 °C for temporary storage and transportation. In this Research Topic, Okuda et al. examined the impact of cycling between these temperatures on the viability of cryopreserved human (h)iPSCs. Temperature fluctuations above the glass transition temperature of −120 °C for dimethyl sulfoxide, the cryoprotectant, trigger a cascade of events that culminate in cell death. Cell damage initiates with an influx of the cryoprotectant that disrupts mitochondrial cytochrome signaling and membrane potential, as well as cell attachment. These findings suggest temperature cycling causes delayed cell death from mitochondrial dysfunction rather than immediate death from loss of cell membrane integrity, as widely accepted. Understanding cellular responses to temperature fluctuations will aid in the development of quality control strategies, such as precise temperature controls and cell assays, to preserve the viability and function of iPSCs throughout the cold chain.

Frequently, iPSC-derived cells exhibit an immature, fetal-like phenotype upon differentiation (Baxter et al., 2015; Yang et al., 2023). Achieving functional maturity remains a critical barrier to the use of iPSCs. Josvai et al. investigated the mechanism by which hiPSC-derived cardiac fibroblasts (hiPSC-CFs) stimulate the contractile function of hiPSC-derived cardiomyocytes (hiPSC-CMs) on a 2D substrate of micropatterned extracellular matrix. Compared with monocultures of hiPSC-CMs, co-cultures of hiPSC-CMs and hiPSC-CFs exhibit larger contractile strain, increased rate of spontaneous contraction, faster kinetics, and increased contractile anisotropy and myofibril alignment. Conditioned medium from hiPSC-CFs is sufficient to improve only a subset of the contractile properties of cardiomyocytes, namely the amplitude and upstroke kinetics of contractile strain. These findings suggest that cardiac fibroblasts drive cardiomyocytes toward functional maturity through a mechanism that requires both paracrine signaling and direct cellular interactions. This research emphasizes the crucial role of cardiac fibroblasts in generating functional cardiomyocytes as cellular therapies and as *in vitro* cultures for disease modeling and drug screening.

In a related study, Gisone et al. enhanced hiPSC-CM maturation by combining co-culture with 3D hydrogels to mimic not only the composition but also the architecture of cardiac tissue. They found that hiPSC-CMs produce higher expression of cardiac maturation markers when co-cultured with human coronary artery endothelial cells in a 3D gelatin methacryloyl hydrogel than when cultured as a classic 2D monoculture. Omics analysis yielded consistent results, which indicate an upregulation of pathways for cardiac differentiation and contraction in the 3D system. An added benefit is the increased cell viability and decreased oxidative stress of the hydrogel co-culture relative to the 2D monoculture. The improved maturation profile of hiPSC-CMs in a biomimetic environment may provide more effective cellular therapies and reliable *in vitro* models.

The clinical success of cellular therapies depends on their ability to localize and survive at the target tissue (Madsen et al., 2020; Xu et al., 2025). Using the novel Antares 2 luciferase reporter, Yuan et al. explored the fate of iPSC-derived mesenchymal stem cells (iPSC-MSCs) after injection into the knee joint cavity of arthritic rats. The reporter is a fusion of the luciferase NanoLuc to the orange fluorescent protein CyOFP, which produces exceptionally bright bioluminescence for sensitive detection in deep tissues during imaging (Yeh et al., 2017). The iPSC-MSCs significantly improved cartilage damage and persisted in the joint cavity without migrating to other sites for over 2 weeks, indicating that therapeutic effects and tissue repair occur locally rather than systemically. Imaging of dissected tissue suggested that iPSC-MSCs injected into the joint cavity are first absorbed by the loose connective tissue of the synovium before distributing to the meniscus and cartilage. This study confirms the therapeutic potential of iPSC-MSCs to treat osteoarthritis and elucidates the underlying mechanism of joint repair.

Extracellular vesicles (EVs) are a compelling cell-free therapy in regenerative medicine that are safer, less immunogenic and logistically superior to direct stem cell use (Goo et al., 2024; Porzionato et al., 2021). A study by Palama et al. compared the aging of hMSCs and hiPSC-MSCs during long-term expansion, along with the anti-inflammatory properties of their respective EVs in an *in vitro* model of osteoarthritis. hMSC expansion leads to cellular senescence, reduced potency, and diminished anti-inflammatory properties of their derived EVs over several passages, consistent with previous reports (Izadpanah et al., 2008). hiPSC-MSCs maintain their stem cell and EV function for a longer duration than conventional hMSCs, suggesting that EVs derived from hiPSC-MSCs offer a wider therapeutic window than those derived from traditional hMSCs. The authors noted, however, variability in the biological properties across different batches of hiPSC-MSCs and their EVs. Further research is crucial to minimize batch-to-batch variability and produce reliable treatment outcomes with hiPSC-derived EV therapies.

This Research Topic showcases current research to achieve the full potential of iPSC technology. Specifically, the featured articles lay the groundwork for quality control to prevent cryoinjury, biomimetic systems to drive functional maturity, a mechanistic understanding of tissue repair, and cell-free therapies derived from extracellular vesicles. The resulting advances will deliver more effective cellular therapies, accurate disease modeling, and streamlined drug development with iPSCs. Through these and other research efforts, the day is rapidly approaching when the extraordinary promise of iPSCs becomes a reality.

## Figures and Tables

**FIGURE 1:**
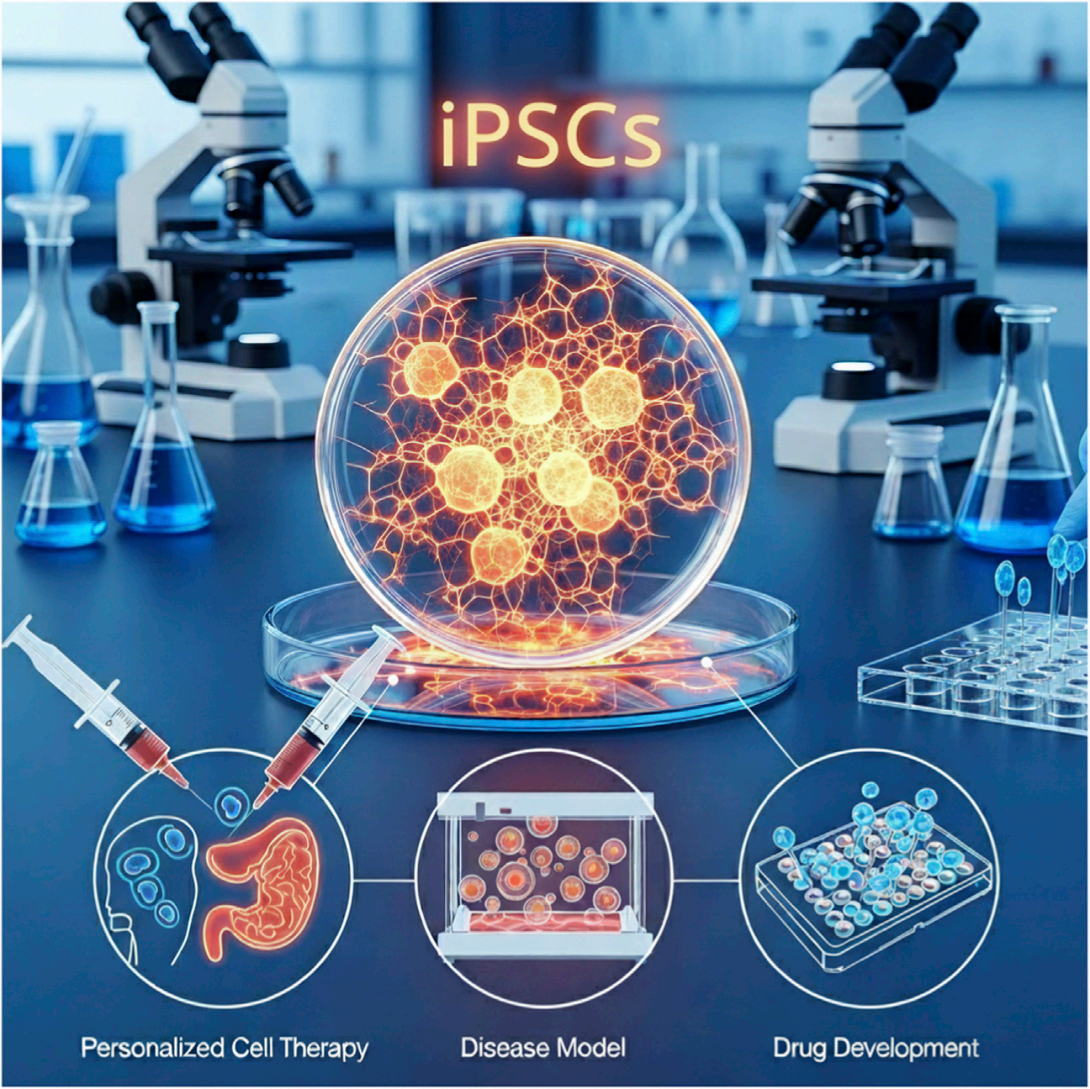
Applications of iPSC technology in personalized cell therapies, disease models and drug development. Image was generated using Whisk, an AI image remixing tool by Google Labs (labs.google/whisk).
